# A Case of Central Precocious Puberty Due to Concomitant Hypothalamic Hamartoma and Juvenile Pilocytic Astrocytoma

**DOI:** 10.4274/jcrpe.1306

**Published:** 2014-09-05

**Authors:** Gönül Çatlı, Ayhan Abacı, Ahmet Anık, Handan Güleryüz, Erdener Özer, İrfan Öcal, Nurullah Yüceer, Kamer Mutafoğlu

**Affiliations:** 1 Dokuz Eylül University Faculty of Medicine, Department of Pediatric Endocrinology, İzmir, Turkey; 2 Dokuz Eylül University Faculty of Medicine, Department of Radiology, İzmir, Turkey; 3 Dokuz Eylül University Faculty of Medicine, Department of Pathology, İzmir, Turkey; 4 İzmir Atatürk Training Research and Hospital, Department of Pathology, İzmir, Turkey; 5 Dokuz Eylül University Faculty of Medicine, Department of Neurosurgery, İzmir, Turkey; 6 Dokuz Eylül University Faculty of Medicine, Department of Pediatric Oncology, İzmir, Turkey

**Keywords:** Hypothalamic hamartoma, pilocytic astrocytoma, Central precocious puberty

## Abstract

Central precocious puberty (CPP) is caused by premature activation of the hypothalamo-pituitary-gonadal axis. More than 50% of boys with CPP have an identifiable etiology. Hypothalamic hamartoma (HH), hydrocephalus, tumors, infections, congenital defects, ischemia, radiation, or injury of the brain are the most common causes of secondary CPP. In this report, we present the case of a 2 years and 9 months old male patient who had a 30x40 mm contrast-enhancing suprasellar mass and was histopathologically diagnosed with giant HH. However, since HHs are designated as non-enhancing masses, considering the possibility of an incomplete diagnosis of a glial tumor, the patient was followed up. Clinical and radiological follow-up revealed stable findings with no evidence of tumor growth until the third year after surgery when he presented with neurological deficit due to the rapid growth of the suprasellar mass. After the second surgery, histopathological examination of the biopsy specimen revealed the lesion to be a juvenile pilocytic astrocytoma (PA). The concomitance of HH and juvenile PA is very rare. To our knowledge, this is the first report of a patient with concomitant juvenile PA and HH who developed CPP and did not have gelastic epilepsy despite the rapidly growing giant mass.

## INTRODUCTION

Central precocious puberty (CPP) or gonadotropin-releasing hormone-dependent precocious puberty, which is caused by premature activation of the hypothalamo-pituitary-gonadal axis is more common in girls than in boys (1). Approximately 95% of girls with CPP have idiopathic CPP and only 5% have an underlying central nervous system (CNS) disorder. On the other hand, more than 50% of boys with CPP have an identifiable etiology and idiopathic CPP is a diagnosis of exclusion (2). Therefore, CNS disease needs to be ruled out before reaching a diagnosis of idiopathic precocious puberty in a male patient. Tumors (optic glioma, astrocytoma, ependymoma, medulloblastoma), infections, congenital defects, cerebral ischemia and radiation or injury to the CNS can all lead to secondary CPP (2). The most common underlying defects are hypothalamic hamartoma (HH) and hydrocephalus (1). The reported incidence of HH in patients with precocious puberty varies from 14% to 58% (3). Juvenile pilocytic astrocytomas (PA) are low-grade CNS tumors which, have a low growth potential and have been reported to regress spontaneously without malignant changes (4,5). Concomitance of HH with juvenile PA is very rare.

In this report, we present the case of a patient with concomitant HH and juvenile PA who developed CPP within the second year of clinical follow-up and who showed no epileptic symptoms despite the giant mass.

## CASE REPORT

**Figure 1:**
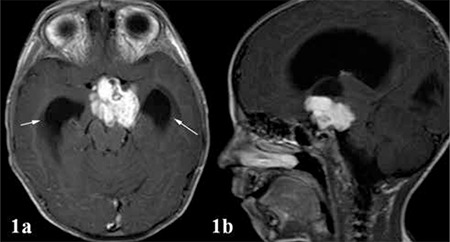
n the T1-weighted axial (a) and sagittal (b) sections a contrast-enhancing suprasellar mass with solid and cystic components is shown. Hydrocephalus is present due to compression by the mass (arrows)

**Figure 2:**
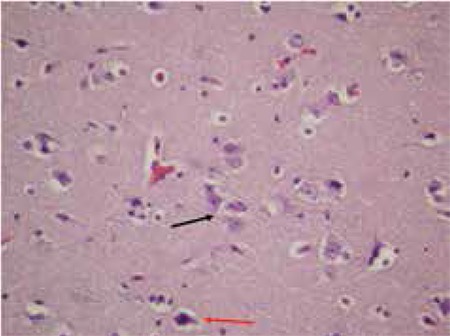
Histological examination disclosed single or clustered hamartomatous neuronal cells (black arrows) as well as reactive astrocytes (red arrow) (H&E, x200)

A 29/12 years old male patient had been admitted to the emergency department with complaints of headache, vomiting and gait disorder which had developed within the past month. In brain magnetic resonance imaging (MRI), a lobulated contoured and contrast-enhancing suprasellar mass was detected. The mass was 30x40 mm in size, consisted of solid and cystic components and had led to hydrocephalus (Figure 1). After incomplete surgical resection of the tumor, the histopathological findings were reported to be compatible with neuronal hamartoma (Figure 2).

The patient was referred to the pediatric endocrinology clinic for evaluation of probable anterior and posterior pituitary hormone deficiencies. On physical examination, his weight was 16 kg (75-90p) [+1.28 standard deviation score (SDS)] and height was 98 cm (90-95p) (+1.36 SDS). Clinical findings were normal and puberty was compatible with Tanner stage 1. Determination of hormone levels in a morning plasma specimen revealed an adrenocorticotropic hormone level of 46 pg/mL (normal, 0-46), a serum cortisol level of 19.7 µg/dL (normal, 0.8-1.9), a prolactin level of 3.19 ng/mL (normal, 2.5-17). Thyroid-stimulating hormone level was 3.98 mIU/mL (normal, 0.4-5) and free thyroxine level was 1.05 ng/dL (normal, 0.8-1.9). In view of the location of the mass, it was decided that the patient needed to be followed up for the possible development of hypothalamo-pituitary disorders such as CPP and growth failure. He was also being followed up clinically and radiologically for his giant intracranial mass. 

**Figure 3:**
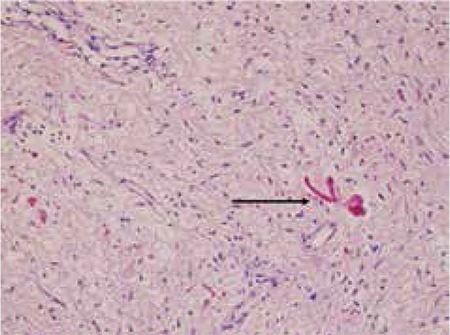
Diffuse astrocytoma predominantly composed of piloid cells and Rosenthal fibers (black arrows) in piloid areas (H&E, X200)

At the age of five years and six months, the patients’ weight was 23 kg (75-90p) (+1.23 SDS), his height was 116 cm (75-90p) (+0.98 SDS) and his testicular volumes were 5 mL and 6 mL. He had no pubic hair. During the physical examination, aggressive behavior and mental retardation were noted. However, he had no history of any type of epilepsy. Biochemical measurements revealed a basal follicle-stimulating hormone level of 2.48 mIU/mL (normal, 0.7-11.1), luteinizing hormone (LH) level of 1.45 mIU/mL (normal, 0.8-7.6) and a total testosterone level of 50 ng/dL (normal, 3-30). Scrotal USG revealed age-inappropriate enlargement of the testicles with normal echotexture - the right testicle was 22x15x35 mm and the left testicle was 21x16x34 mm in diameter. The bone age was compatible with the chronological age. In LH-releasing hormone test, a peak LH value of 15 mlU/mL (>5) was detected by direct chemiluminometric technique. The patient was diagnosed with CPP and started on gonadotropin-releasing hormone (GnRH) agonist treatment (triptorelin acetate 3.75 mg every 4 weeks). A normal electroencephalogram ruled out subclinical epileptic activity. Until the third year after the surgery, the patient was clinically and radiologically stable with no evidence of tumor growth. However, at the age of six years and three months, the patient was referred with neurological deficit due to the rapid growth of the suprasellar mass. After the second surgery, he died because of brain herniation. Histopathological examination of the biopsy specimen revealed the lesion to be a juvenile PA (Figure 3).

## DISCUSSION

HHs are rare (1:200 000) heterotopic congenital intracranial malformations with a mean diameter of 17.9-18 mm. They consist of benign neural and glial cells and usually originate from tuber cinereum, mamillary bodies and posterior hypothalamus (6,7,8). HHs are divided into two groups according to their MRI findings: i) parahypothalamic (pedunculated) type which is generally related to CPP and ii) HHs (intrahypothalamic) which are usually associated with epileptic activities (9). The most frequently reported clinical findings in HH are CPP (63%), gelastic epilepsy (61%) and in 23% of cases, a combination of CPP and gelastic epilepsy (6). In a review of MRI findings and clinical features, 8 out of 10 patients with HH were found to have developed CPP before the age of 3 years (2). The current patient presented with neurological symptoms due to a suprasellar mass, which was initially diagnosed as giant HH according to the histopathological examination results. Two years after incomplete resection of the mass, when he was 5.5 years old, CPP developed.

The treatment of choice in HH is surgical; however, due to adhesion and invasion to the adjacent structures, it cannot be usually removed completely. In HH cases with intractable epilepsy and mass-related clinical findings, while surgical resection is recommended, wide resections must be avoided to prevent the development of neurologic sequelae (6,10). In cases without neurological findings, non-invasive treatment methods such as radiofrequency ablation and radio-surgical treatment are also recommended (6). Most cases with HH-related CPP respond well to the GnRH analog treatment. In the present case, because of presence of neurological findings, surgical intervention was performed, but the mass could not be removed completely. GnRH analog treatment was given for the CPP and progression of puberty was clinically and biochemically restrained with this treatment.

PA is the most common benign brain tumor in childhood. It usually arises within the cerebellum and rarely in the hypothalamic/chiasmatic region, but it may occur in any area where astrocytes are present (11,12,13,14). PAs are often cystic and characteristically contrast-enhancing tumors in computed tomography and MRI studies (15). The prognosis for children with PA is generally good and the 20-year survival rate is approximately 70%-80% even after undergoing subtotal resection (13). However, malignant transformation, leptomeningeal seeding or recurrence can also be encountered in these tumors (11,16). There is no report documenting spontaneous malignant transformation of a WHO grade 1 PA. A review of the literature showed that individuals who suffered malignant transformation all had tumors that had been previously irradiated (4). 

CPP due to astrocytoma has been reported previously (17). The current patient had a 30x40 mm contrast-enhancing suprasellar mass, which consisted of solid and cystic components and caused hydrocephalus demonstrated on brain MRI. After incomplete surgical resection of the tumor, based on histopathological findings, he was initially diagnosed with giant HH. However, since HHs are designated as non-enhancing masses (15), considering the possibility of an incomplete diagnosis of a glial tumor, the patient underwent clinical and radiological follow-up which revealed stable findings with no evidence of tumor growth until the third year after the surgery. However, at the age of six years and three months, he presented with neurological deficit due to the rapid growth of the suprasellar mass. After the second surgery, histopathological examination of the biopsy specimen revealed the lesion to be a juvenile PA. We suggest that in this patient, the limited resection of the mass in the first surgical intervention may have led to an incomplete diagnosis. Juvenile PA and HH are pathologically quite different lesions. However, they behave similarly in that neither tends to show spontaneous malignant transformation and even large PAs have been shown to regress without treatment (4,18). However, a small subset of children with this tumor experience a poor clinical course with shorter disease-free survival and higher mortality rates. Although it has long been recognized that particular PAs behave aggressively, the reasons for this have not yet been elucidated (13).

Previously, Park et al (19) have reported a 6-year-old girl presenting with intractable gelastic seizures with a spontaneously regressing juvenile PA and a concomitant HH. To our knowledge, this is the second report of a patient with concomitant juvenile PA and HH. Distinct from the previous report, our patient developed CPP and despite the rapidly growing giant mass he did not have gelastic epilepsy.

In conclusion, although HH is the most frequent organic cause of CPP, in a patient with a hypothalamic mass, the presence of pathological contrast enhancement in MRI should alert the physician to consider a diagnosis other than HH, particularly a glial tumor. With this case report, we would like to emphasize that although rare, HH and juvenile PA may occur concurrently in a patient.
